# Triple-negative breast cancer survival prediction: population-based research using the SEER database and an external validation cohort

**DOI:** 10.3389/fonc.2024.1388869

**Published:** 2024-06-10

**Authors:** Yu Qiu, Yan Chen, Haoyang Shen, Shuixin Yan, Jiadi Li, Weizhu Wu

**Affiliations:** The Affiliated Lihuili Hospital, Ningbo University, Ningbo, China

**Keywords:** triple-negative breast cancer, nomogram, SEER database, overall survival, cancer-specific survival

## Abstract

**Introduction:**

Triple-negative breast cancer (TNBC) is linked to a poorer outlook, heightened aggressiveness relative to other breast cancer variants, and limited treatment choices. The absence of conventional treatment methods makes TNBC patients susceptible to metastasis. The objective of this research was to assess the clinical and pathological traits of TNBC patients, predict the influence of risk elements on their outlook, and create a prediction model to assist doctors in treating TNBC patients and enhancing their prognosis.

**Methods:**

We included 23,394 individuals with complete baseline clinical data and survival information who were diagnosed with primary TNBC between 2010 and 2015 based on the SEER database. External validation utilised a group from The Affiliated Lihuili Hospital of Ningbo University. Independent risk factors linked to TNBC prognosis were identified through univariate, multivariate, and least absolute shrinkage and selection operator regression methods. These characteristics were chosen as parameters to develop 3- and 5-year overall survival (OS) and breast cancer-specific survival (BCSS) nomogram models. Model accuracy was assessed using calibration curves, consistency indices (C-indices), receiver operating characteristic curves (ROCs), and decision curve analyses (DCAs). Finally, TNBC patients were divided into groups of high, medium, and low risk, employing the nomogram model for conducting a Kaplan-Meier survival analysis.

**Results:**

In the training cohort, variables such as age at diagnosis, marital status, grade, T stage, N stage, M stage, surgery, radiation, and chemotherapy were linked to OS and BCSS. For the nomogram, the C-indices stood at 0.762, 0.747, and 0.764 in forecasting OS across the training, internal validation, and external validation groups, respectively. Additionally, the C-index values for the training, internal validation, and external validation groups in BCSS prediction stood at 0.793, 0.755, and 0.811, in that order. The findings revealed that the calibration of our nomogram model was successful, and the time-variant ROC curves highlighted its effectiveness in clinical settings. Ultimately, the clinical DCA showcased the prospective clinical advantages of the suggested model. Furthermore, the online version was simple to use, and nomogram classification may enhance the differentiation of TNBC prognosis and distinguish risk groups more accurately.

**Conclusion:**

These nomograms are precise tools for assessing risk in patients with TNBC and forecasting survival. They can help doctors identify prognostic markers and create more effective treatment plans for patients with TNBC, providing more accurate assessments of their 3- and 5-year OS and BCSS.

## 1 Introduction

In the realm of female cancers, breast cancer ranks among the highest in terms of fatality rates, with 2.3 million new cases (11.7%) and 685,000 fatalities (6.9%) anticipated in 2020 (1). In triple-negative breast cancer (TNBC), the oestrogen and progesterone receptors (ER/PR) and the human epidermal growth factor receptor 2 (HER2) are not expressed (2). Because ER, PR, and HER2 are not expressed, the prognosis is poor, few therapeutic choices exist, and both conventional targeted and endocrine therapies are ineffective (3). Differing from other variants of breast cancer, TNBC has a higher 5-year mortality rate, is more invasive, has a poorer prognosis, and recurrence peaks within the first 3 years of diagnosis. Studies suggest that approximately 50% of patients will experience distant metastases, with the chance of long-distance metastases peaking 3 years after surgery. The lungs and liver are the most common metastatic locations, with the bone being less prevalent (4, 5). Patients have improved survival rates and survive longer after treatment, owing to considerable advancements in TNBC treatment in recent years (6, 7). Given the high degree of heterogeneity associated with TNBC, individualised treatment is strongly recommended. Convenient collection of clinicopathological parameters of patients with TNBC to reliably determine the predictive survival time would be an invaluable resource for patients’ families and clinicians.

Many years have passed since the American Joint Committee on Cancer’s (AJCC) tumour, node, and metastasis (TNM) prognostic staging system was first introduced to determine the prognosis of patients with breast cancer. The importance of traditional tumour staging methods has gradually decreased with the development of molecular subtyping and precision therapies. Therefore, depending on this data does not adequately evaluate the likelihood and future outlook of distant metastasis in TNBC patients (8, 9). Literature on prognostic models for TNBC is currently scarce, particularly regarding the poor understanding of tumour-specific survival in patients with TNBC. Nomograms are recognised in the fields of oncology and medicine as common statistical visualisation tools and crucial components of contemporary medical decision-making. They incorporate a variety of factors that impact prognosis and survival and then visualise and quantify these influencing factors. Nomograms can objectively show the results to achieve the effect of accurately predicting the prognosis and survival duration of patients (10, 11).

This research analysed and identified key predictive elements for breast cancer in individuals with TNBC, developing a visual nomogram using data from the SEER database and patient records from our hospital. The objectives of this study were to improve the accuracy of survival prediction, lay the groundwork for creating customised treatment plans, and analyse the prognostic characteristics of female patients with TNBC.

## 2 Materials and methods

### 2.1 Sources of data and patient selection

The study utilised a database known as SEER, which was released in November 2022. Patients included in the study were selected from SEER*Stat Version 8.4.2, a database containing information on population, patient characteristics, tumour features, diagnosis, and healthcare information from 17 cancer registries, representing around 28% of cancer cases in the United States from 2000 to 2020. A signed SEER research data agreement form was submitted to the SEER programme to access and analyse the SEER database for our study (https://seer.cancer.gov/). Considering the compulsory reporting of cancer in every state across the country, obtaining patient informed consent is not essential for accessing the SEER database.

An analysis of breast cancer patients conducted from January 1, 2010 to December 31, 2015 was performed based on these inclusion criteria (1): female patients aged 18 to 80; (2) initial diagnosis of primary breast cancer; and (3) confirmation of the triple-negative molecular subtype by pathological analysis (ER-/PR-/HER2-). The exclusion criteria included: (1) patients with bilateral and inflammatory breast cancers; (2) patients with multiple primary tumours; (3) absence of critical data such as stage and grade; and (4) patients with missing or incomplete follow-up information. The data entered into the case table included age at diagnosis, race, marital status, histological type, grade, AJCC stage, TNM classification, tumour size, cancer treatment, and other relevant factors. After analysis, participants who lacked information on clinical features or survival were excluded from the final dataset, which included 23,394 patients with TNBC.

The age classification in this manuscript follows the 2023 United Nations World Health Organization guidelines, which are based on assessments of global human body quality and average life expectancy.Accordingly, individuals aged 18–44 years old are considered young adults; those aged 45-59 years old are categorized as middle-aged; and individuals over 60 years old are classified as older adults. Numerous studies have established that morphological assessment of differentiation can yield valuable prognostic insights for breast cancer. Specifically, grade I tumours were well differentiated, grade II moderately differentiated, grade III poorly differentiated, and grade IV undifferentiated. It has been documented that for certain anatomical sites, grades III and IV may be amalgamated into a single grade, a categorization that is applicable to breast cancer.

To validate the proposed nomogram, 230 patients were recruited from The Affiliated Lihuili Hospital of Ningbo University between 2016 and 2020 (**Supplementary Table 1**). The selection of patients for this external validation group adhered to the same criteria for inclusion and exclusion as those used for the training group. The visiting physician conducted follow-ups at regular intervals after the patient was diagnosed with breast cancer: every 6 months for 5 years, annually for 5 years, and finally, when the patient passed away. The deadline for collecting the follow-up data was 31 December, 2023. Reviewing medical histories and conducting phone interviews were two methods used to collect additional data. The patients’ hospital records were initially checked; if the medical records lacked pertinent information, a telephone interview was conducted. The work conducted in this retrospective analysis did not require review by an Institutional Review Board because the patient data were anonymised.

### 2.2 Outcome measurement

The overall survival rate (OS) was determined either by tracking the duration from diagnosis to death from any cause or, in cases where the patient survived, by tracking the period from diagnosis to the final follow-up. The duration of a patient’s survival specific to breast cancer (BCSS) was quantified from the time of diagnosis until their demise from breast cancer, treating other death causes as simultaneous risks.

### 2.3 Statistical analysis

The training and internal validation cohorts were randomly divided according to a 7:3 ratio using SPSS statistics (IBM Corporation, Armonk, NY, USA). We are confident that a ratio of 7 to 3 was the suitable choice for this research. Ensuring the model’s precision involves utilising a significant portion of the data for constructing the nomogram and a smaller portion for validation to prevent overfitting. A chi-squared test was utilised to evaluate the fundamental traits of the cohort groups engaged in training, internal validation, and external validation.

Conducting independent risk factor screening involved using univariate Cox analysis, whereas multivariate Cox regression models estimated hazard ratios and 95% confidence intervals (CIs) to examine independent prognostic factors, confirming the significance of each component in survival. Additionally, we employed 10-fold cross-validation along with least absolute shrinkage and selection operator (LASSO) regression to prevent overfitting of the model. To provide clinicians with a quantifiable tool for assessing OS and BCSS in patients with TNBC, these factors were chosen as prognostic model parameters, and nomograms of OS and BCSS were created using R (version 4.2.0; Vienna, Austria) software.

The prognostic model’s capacity for prediction was assessed using two validation cohorts: one for internal validation and the other for external validation. The “rms” function was used to calculate the concordance index (C-index), which was used to assess the probability that the actual and predicted results were consistent. Receiver operating characteristic (ROC) curves were used to evaluate the prognostic model’s capacity to discriminate, as well as to estimate its sensitivity and specificity. The calibration curve was created using the 1,000 bootstrap sample approach to assess the prediction model’s accuracy. The prediction model’s flexibility and clinical applicability were evaluated using a decision curve analysis (DCA). Finally, to guide the Kaplan-Meier survival analysis, the risk score and X-tile software (version 3.6.1; Yale University, New Haven, CT) were used to categorise all patients into low-, intermediate-, and high-risk groups.

The statistical analysis above was conducted using R software. R packages such as “Survival,” “forestplot,” “Glmnet,” “rms,” “stdca.R,” “survivalROC,” and “survivalminer” (http://www.r-project.org/) were utilised. P < 0.05 was considered statistically significant.

## 3 Results

### 3.1 Baseline features of the study population

**Figure 1:**
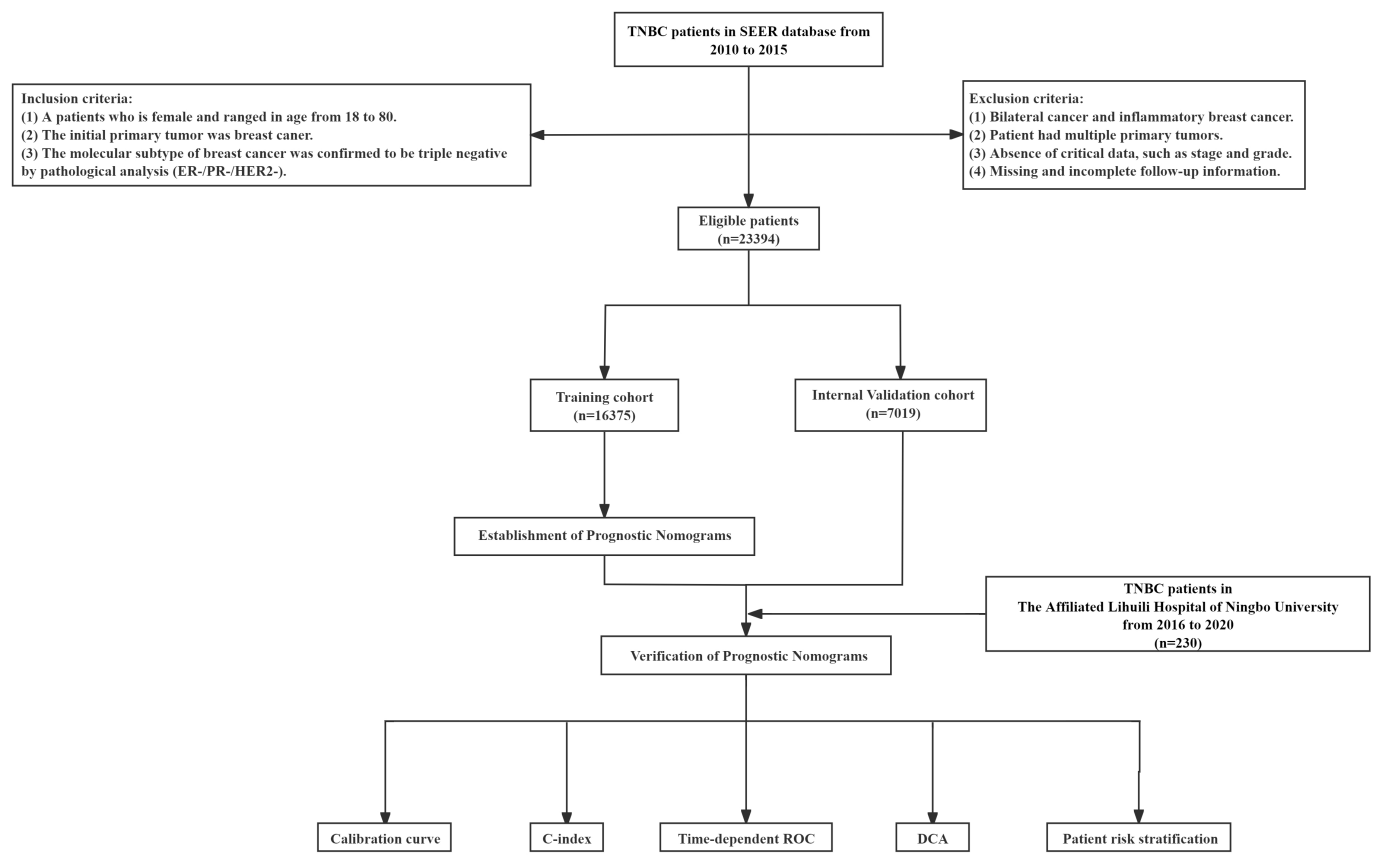
Flowchart of participant inclusion and exclusion.

The SEER database was used to enrol 23,394 eligible patients with TNBC. Patients who met the inclusion criteria but not the exclusion criteria were split at random into two groups: an internal validation cohort (n = 7019) and a training cohort (n = 16375) using a ratio of 7:3. Under the same conditions, 230 patients with TNBC were recruited for external validation from the Affiliated Lihuili Hospital of Ningbo University. **Figure 1** displays a flowchart of the patient screening and research design.

Overall, no significant differences were found (P > 0.05) between the internal validation and training cohorts. In contrast, the SEER cohort differed substantially from the external validation cohort with respect to baseline demographic and treatment data, which may be attributable to differences in geography, race, and treatment schedules. Brief descriptions of the demographics and clinical characteristics of the patients are provided in **Table 1**. The 3- and 5-year overall survival rates were 79.4% and 74.4%, respectively. The 3- and 5-year survival specific to breast cancer rates were 82.5% and 79%, respectively. The median follow-up time was 79 months (interquartile range: 55 months, 104 months).

**Table 1:** Demographic and clinical characteristics of patients with triple-negative breast cancer.

Characteristic	Training cohort (n = 16375)	Internal validation cohort (n =7019)	External validation cohort (n = 230)	P value ^a^	P value ^b^
Age (years)				0.226	0.022
20-44	3111 (19.00)	1342 (19.12)	36 (15.65)		
45-59	6645 (40.58)	2767 (39.42)	114 (49.57)		
60	6619 (40.42)	2910 (41.46)	80 (34.78)		
Race				0.258	<.001
White	11770 (71.88)	5122 (72.97)	0		
Black	3259 (19.90)	1362 (19.40)	0		
AI/AN ^c^	94 (0.57)	42 (0.60)	0		
Asian	1252 (7.65)	493 (7.02)	230 (100)		
Marital status				0.528	<.001
Married	9668 (59.04)	4113 (58.60)	225 (97.83)		
Not married	6707 (40.96)	2906 (41.40)	5 (2.17)		
Histology				0.577	0.162
IDC	14160 (86.47)	6073 (86.52)	209 (90.87)		
ILC	161 (0.98)	59 (0.84)	1 (0.43)		
Other	2054 (12.54)	887 (12.64)	20 (8.70)		
Grade				0.071	0.094
I	340 (2.08)	114 (1.62)	8 (3.48)		
II	2715 (16.58)	1172 (16.70)	46 (20.00)		
III+IV	13320 (81.34)	5733 (81.68)	176 (76.52)		
T-stage				0.385	<.001
T1	7000 (42.75)	3011 (42.90)	129 (56.09)		
T2	6987 (42.67)	2943 (41.93)	90 (39.13)		
T3	1410 (8.61)	650 (9.26)	10 (4.35)		
T4	978 (5.97)	415 (5.91)	1 (0.43)		
N-stage				0.879	0.001
N0	10489 (64.05)	4522 (64.43)	170 (73.91)		
N1	4108 (25.09)	1755 (25.00)	36 (15.65)		
N2	999 (6.10)	410 (5.84)	19 (8.26)		
N3	779 (4.76)	332 (4.73)	5 (2.17)		
M-stage				0.916	0.010
M0	15608 (95.32)	6688 (95.28)	228 (99.13)		
M1	767 (4.68)	331 (4.72)	2 (0.87)		
Surgery				0.492	<.001
Breast-conserving surgery	7945 (48.52)	3346 (47.67)	102 (44.35)		
Mastectomy	7331 (44.77)	3195 (45.52)	128 (55.65)		
No	1099 (6.71)	478 (6.81)	0 (0.00)		
Radiation				0.534	<.001
Yes	8448 (51.59)	3590 (51.15)	106 (46.09)		
No/unknown	7927 (48.41)	3429 (48.85)	124 (53.91)		
Chemotherapy				0.282	0.003
Yes	13190 (80.55)	5611 (79.94)	204 (88.70)		
No/unknown	3185 (19.45)	1408 (20.06)	26 (11.30)		

^a^ Training cohort vs. Internal validation cohort; ^b^ Training cohort vs. External validation cohort.

^C^ American Indian/Alaska Native.

### 3.2 Baseline characteristic comparison and feature selection

**Figure 2:**
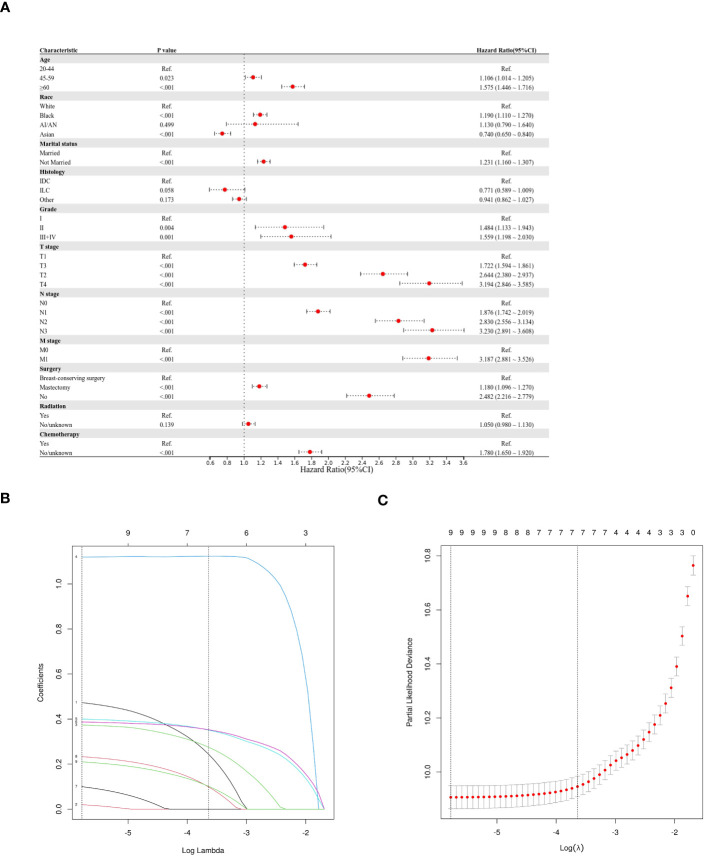
Predictor screening of OS **(A)** Forest map (univariate analysis); **(B, C)** LASSO Cox regression model construction. LASSO coefficients of seventeen features and Selection of tuning parameter (k) for the LASSO model.

**Figure 3:**
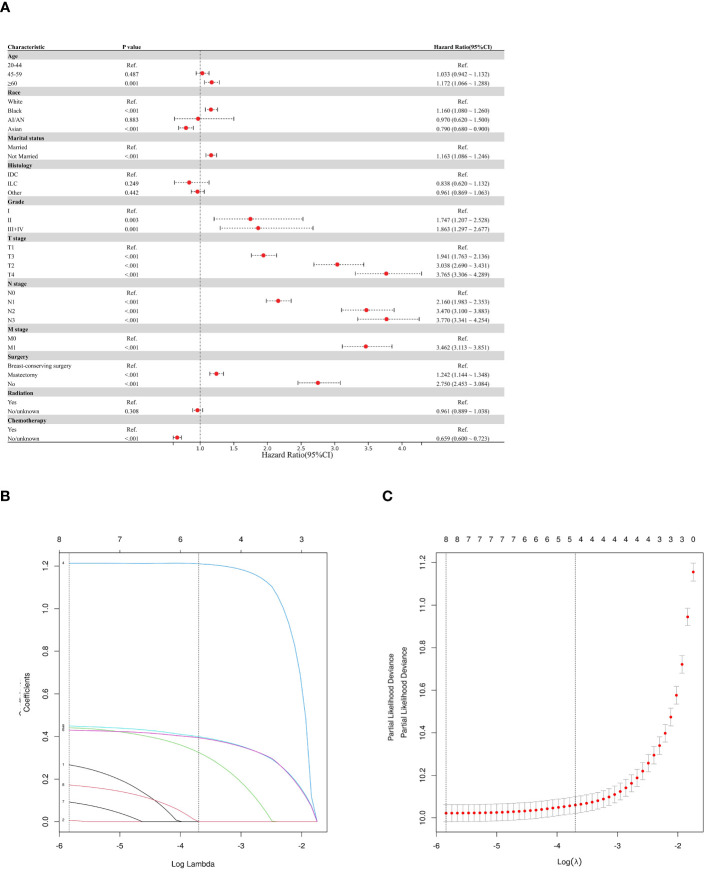
Predictor screening of BCSS **(A)** Forest map (univariate analysis); **(B, C)** LASSO Cox regression model construction. LASSO coefficients of seventeen features and Selection of tuning parameter (k) for the LASSO model.

This study employed three methodologies to find independent predictors of OS and BCSS among individuals with TNBC based on the training cohort data. We examined prognosis-related factors using the Cox proportional hazards regression method. The training cohort comprised 11 clinical parameters. A significant (P < 0.05) association was found in the results from the univariate Cox regression analysis between the clinical parameters and the difference between BCSS and OS (**Table 2**). These statistically significant covariates were then incorporated in the multivariate Cox regression (**Table 3**), and the results were presented as forest plots (**Figures 2A**, **3A**), where a total of nine (9/11) factors were selected (P < 0.05). In the LASSO regression analysis, we also evaluated variables that had prognostic significance in the univariate analysis. Among these, nine variables (9/11) had a statistically significant association with OS (**Figures 2B, C**), whereas eight factors (8/11) had a statistically significant relationship with BCSS (**Figures 3B, C**) (P < 0.05).

**Table 2:** Univariate analysis of the triple-negative breast cancer patients for overall and cancer-specific survival.

Variables	BCSS		OS	
	HR (95% CI)	P value	HR (95% CI)	P value
Age (years)				
20-44	Ref.		Ref.	
45-59	0.946 (0.863 ~ 1.036)	0.231	1.021 (0.937 ~ 1.112)	0.641
60	0.957 (0.873 ~ 1.048)	0.340	1.358 (1.250 ~ 1.475)	**<.001**
Race				
White	Ref.		Ref.	
Black	1.351 (1.250 ~ 1.460)	**<.001**	1.340 (1.250 ~ 1.440)	**<.001**
AI/AN	1.050 (0.670 ~ 1.630)	0.841	1.150 (0.800 ~ 1.660)	0.451
Asian	0.820 (0.710 ~ 0.940)	0.005	0.750 (0.660 ~ 0.850)	**<.001**
Marital status				
Married	Ref.		Ref.	
Not married	1.417 (1.326 ~ 1.514)	**<.001**	1.487 (1.403 ~ 1.575)	**<.001**
Histology				
IDC	Ref.		Ref.	
ILC	1.403 (1.045 ~ 1.883)	0.024	1.319 (1.013 ~ 1.717)	0.040
Other	1.050 (0.950 ~ 1.160)	0.340	1.045 (0.958 ~ 1.140)	0.320
Grade				
I	Ref.		Ref.	
II	2.315 (1.602 ~ 3.345)	**<.001**	1.692 (1.295 ~ 2.211)	**<.001**
III+IV	2.743 (1.914 ~ 3.930)	**<.001**	1.860 (1.435 ~ 2.410)	**<.001**
T-stage				
T1	Ref.		Ref.	
T2	2.535 (2.314 ~ 2.776)	**<.001**	1.963 (1.826 ~ 2.111)	**<.001**
T3	6.294 (5.641 ~ 7.024)	**<.001**	4.356 (3.967 ~ 4.784)	**<.001**
T4	13.641 (12.244 ~ 15.190)	**<.001**	9.095 (8.282 ~ 9.989)	**<.001**
N-stage				
N0	Ref.		Ref.	
N1	3.297 (3.047 ~ 3.567)	**<.001**	2.480 (2.320 ~ 2.652)	**<.001**
N2	5.756 (5.181 ~ 6.395)	**<.001**	4.108 (3.738 ~ 4.515)	**<.001**
N3	9.691 (8.729 ~ 10.76)	**<.001**	6.779 (6.164 ~ 7.456)	**<.001**
M-stage				
M0	Ref.		Ref.	
M1	13.264 (12.151 ~ 14.479)	**<.001**	10.747 (9.885 ~ 11.683)	**<.001**
Surgery				
Breast-conserving surgery	Ref.		Ref.	
Mastectomy	2.105 (1.950 ~ 2.272)	**<.001**	1.782 (1.671 ~ 1.900)	**<.001**
No	8.308 (7.520 ~ 9.177)	**<.001**	6.357 (5.813 ~ 6.953)	**<.001**
Radiation				
Yes	Ref.		Ref.	
No/unknown	1.230 (1.150 ~ 1.310)	**<.001**	1.250 (1.180 ~ 1.330)	**<.001**
Chemotherapy				
Yes	Ref.		Ref.	
No/unknown	0.890 (0.810 ~ 0.970)	0.008	1.230 (1.140 ~ 1.310)	**<.001**

Bold indicates that P is less than 0.01, indicating statistical significance.

**Table 3:** Multivariate Cox proportional hazard model of breast cancer-specific survival (BCSS) and overall survival (OS) in all patients.

Variables	BCSS		OS	
	HR (95% CI)	P value	HR (95% CI)	P value
Age (years)				
20-44	Ref.		Ref.	
45-59	1.033 (0.942 ~ 1.132)	0.487	1.106 (1.014 ~ 1.205)	0.023
60	1.172 (1.066 ~ 1.288)	0.001	1.575 (1.446 ~ 1.716)	**<.001**
Race				
White	Ref.		Ref.	
Black	1.160 (1.080 ~ 1.260)	**<.001**	1.190 (1.110 ~ 1.270)	**<.001**
AI/AN	0.970 (0.620 ~ 1.500)	0.883	1.130 (0.790 ~ 1.640)	0.499
Asian	0.790 (0.680 ~ 0.900)	**<.001**	0.740 (0.650 ~ 0.840)	**<.001**
Marital status				
Married	Ref.		Ref.	
Not married	1.163 (1.086 ~ 1.246)	**<.001**	1.231 (1.160 ~ 1.307)	**<.001**
Histology				
IDC	Ref.		Ref.	
ILC	0.838 (0.620 ~ 1.132)	0.249	0.771 (0.589 ~ 1.009)	0.058
Other	0.961 (0.869 ~ 1.063)	0.442	0.941 (0.862 ~ 1.027)	0.173
Grade				
I	Ref.		Ref.	
II	1.747 (1.207 ~ 2.528)	0.003	1.484 (1.133 ~ 1.943)	0.004
III+IV	1.863 (1.297 ~ 2.677)	0.001	1.559 (1.198 ~ 2.030)	0.001
T-stage				
T1	Ref.		Ref.	
T2	1.941 (1.763 ~ 2.136)	**<.001**	1.722 (1.594 ~ 1.861)	**<.001**
T3	3.038 (2.690 ~ 3.431)	**<.001**	2.644 (2.380 ~ 2.937)	**<.001**
T4	3.765 (3.306 ~ 4.289)	**<.001**	3.194 (2.846 ~ 3.585)	**<.001**
N-stage				
N0	Ref.		Ref.	
N1	2.160 (1.983 ~ 2.353)	**<.001**	1.876 (1.742 ~ 2.019)	**<.001**
N2	3.470 (3.100 ~ 3.883)	**<.001**	2.830 (2.556 ~ 3.134)	**<.001**
N3	3.770 (3.341 ~ 4.254)	**<.001**	3.230 (2.891 ~ 3.608)	**<.001**
M-stage				
M0	Ref.		Ref.	
M1	3.462 (3.113 ~ 3.851)	**<.001**	3.187 (2.881 ~ 3.526)	**<.001**
Surgery				
Breast-conserving surgery	Ref.		Ref.	
Mastectomy	1.242 (1.144 ~ 1.348)	**<.001**	1.180 (1.096 ~ 1.270)	**<.001**
No	2.750 (2.453 ~ 3.084)	**<.001**	2.482 (2.216 ~ 2.779)	**<.001**
Radiation				
Yes	Ref.		Ref.	
No/unknown	0.961 (0.889 ~ 1.038)	0.308	1.050 (0.980 ~ 1.130)	0.139
Chemotherapy				
Yes	Ref.		Ref.	
No/unknown	0.659 (0.600 ~ 0.723)	**<.001**	1.780 (1.650 ~ 1.920)	**<.001**

Bold indicates that P is less than 0.01, indicating statistical significance.

### 3.3 Prognostic nomogram for patients diagnosed with TNBC

**Figure 4:**
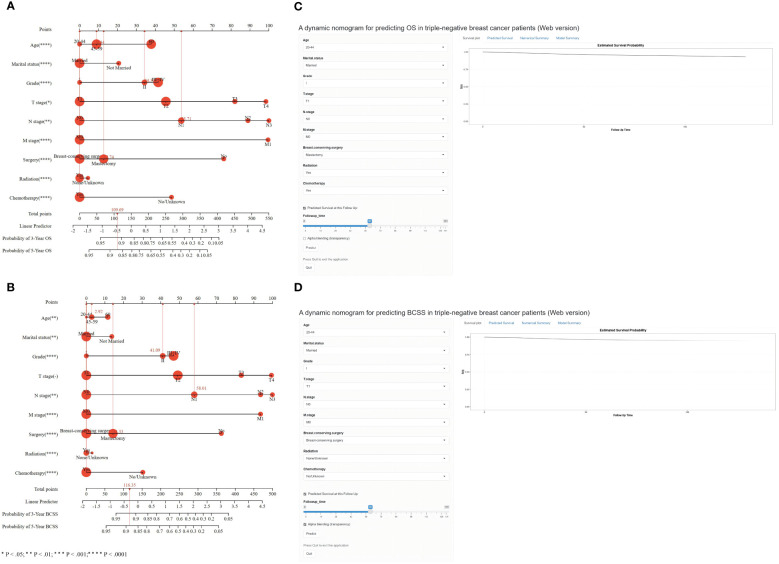
Nomogram for predicting 3- and 5-year OS **(A)** and BCSS **(B)** of TNBC patients with independent prognostic factors. Dynamic nomogram of predicting OS **(C)** and BCSS **(D)** in TNBC patients.

We finally included the nine characteristics (age at diagnosis, marital status, grade, T stage, N stage, M stage, surgery, radiation treatment, and chemotherapy) in the survival nomogram of patients with TNBC in the training cohort by combining the findings of the multivariate Cox and LASSO regression analyses with the conclusions of previous clinical retrospective studies to forecast their BCSS and 3- and 5-year OS (**Figure 4**). As shown in **Figure 4A**, the most significant indicators of OS were the AJCC TNM stage, grade, surgical status, and whether chemotherapy was used. Similarly, **Figure 4B** demonstrates that AJCC TNM stage, grade, surgical status, and whether chemotherapy was administered were the most significant predictors of BCSS.

Every variable was given a unique point on a scale to use the nomogram. The sum of the points for each variable determines the overall point total for a single patient. The prognosis of patients with TNBC with higher scores was worse than that of patients with lower scores. When the total score is projected onto the maximum score table of the nomogram, the likelihood of OS and BCSS at 3 and 5 years in patients can be predicted.

An example of the applicable usefulness of the nomograms comprising all significant characteristics was provided by one normal patient with TNBC. Following mastectomy, a 45-year-old who had TNM stage T1N1M0 underwent chemotherapy and radiation therapy. The patient received 109.69 points on the OS nomogram, corresponding to probabilities of 0.91 and 0.87, respectively, over the following 3 and 5 years. The 3- and 5-year OS rates, or around 0.92 and 0.89, respectively, were correlated with the overall score of 116.35 for all BCSS factors.

To help doctors adopt an easy-to-use web-based interface, we created a dynamic nomogram in addition to the simple one (**Figures 4C, D**). The values of the nine predictor variables may be readily entered, allowing the survival probability and 95% CI to be exported to the right side of the interface by clicking the “Predict” button (https://os-tnbc.shinyapps.io/dynnomapp/) (https://bcss-tnbc.shinyapps.io/dynnomapp/).

### 3.4 Performance and validation of the nomogram

External and internal validations were conducted on the nomogram using the training and validation cohorts.

By computing Harrell’s C-index, the accuracy of the final nomogram’s prediction was ascertained. For OS and BCSS, respectively, the C-index values of the nomograms in the training cohort were 0.762 (95% CI, 0.755–0.770) and 0.793 (95% CI, 0.785–0.801). The C-index values for OS and BCSS in the validation cohort were 0.747 (95% CI, 0.736–0.759) and 0.775 (95% CI, 0.763–0.788), respectively. The external validation cohort’s C-index values were 0.764 (95% CI, 0.671–0.856) for OS and 0.811 (95% CI, 0.734–0.889) for BCSS. All of these exceeded the expected 0.7 for a system capable of accurately predicting both OS and BCSS.

**Figure 5:**
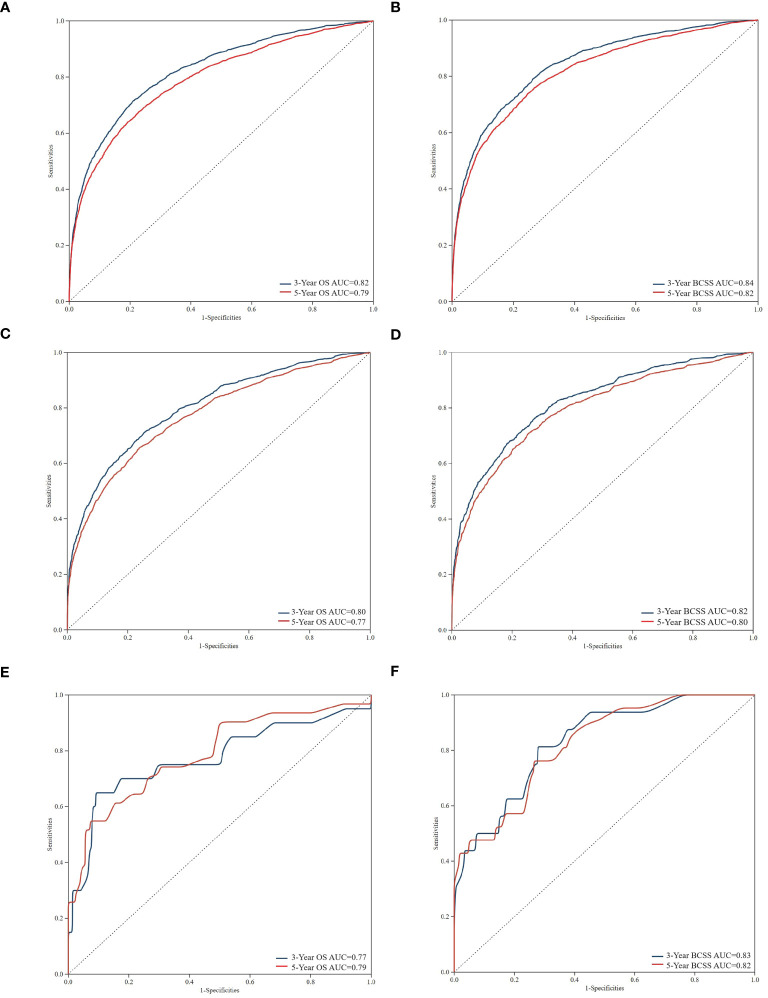
Time-dependent receiver operating characteristic (ROC) curves for assessing the discrimination of the condition survival nomogram in training cohort **(A, B)**, internal validation cohort **(C, D)**, and external validation cohort **(E, F)**, respectively. AUC, area under the curve.

Using ROC curves, we assessed the efficacy of the nomogram in detail. Time-dependent ROCs at 3 and 5 years demonstrated that this model was adequately differentiable. **Figures 5A, C, E** display the areas under the curves (AUCs) for OS [training cohort: 3-year OS 0.82 (95% CI, 0.83–0.82); 5-year OS 0.79 (95% CI, 0.80–0.78); internal validation cohort: 3-year OS 0.80 (95% CI, 0.82–0.79); 5-year OS 0.77 (95% CI, 0.79–0.76); external validation cohort: 3-year OS 0.78 (95% CI, 0.91–0.64); and 5-year OS 0.79 (95% CI, 0.89–0.69)] whereas **Figures 5B, D, F** illustrate the 3-, and 5-year values of the AUC regarding the nomogram for BCSS [training cohort: 3-year BCSS 0.84 (95% CI, 0.85–0.83); 5-year BCSS 0.82 (95% CI, 0.83–0.81); internal validation cohort: 3-year BCSS 0.82 (95% CI, 0.84–0.81); 5-year BCSS 0.80 (95% CI, 0.81–0.78); external validation cohort: 3-year BCSS 0.83 (95% CI, 0.93–0.74); and 5-year BCSS 0.82 (95% CI, 0.91–0.73)]. When the AUC value was larger and the ROC curve was closer to the upper-left corner, the prediction model performed better in discriminating cases.

**Figure 6:**
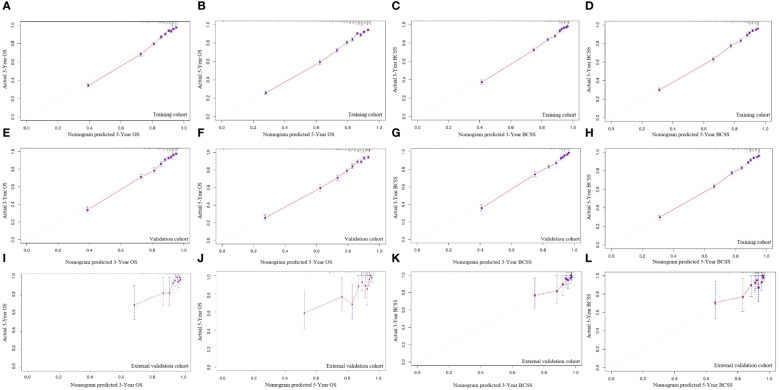
The calibration curves for predicting OS and BCSS at 3-year and 5-year in the training cohort **(A–D)**, and in the internal validation cohort **(E–H)**, and in the external validation cohort **(I–L)**.

**Figure 6** displays the survival nomogram’s calibration curve, which indicates that the actual and nomogram-predicted survival rates agree well. Training, internal validation, and external validation calibration plots all showed strong predictive performance of the model, nearing the ideal 45° line in all cases.

**Figure 7:**
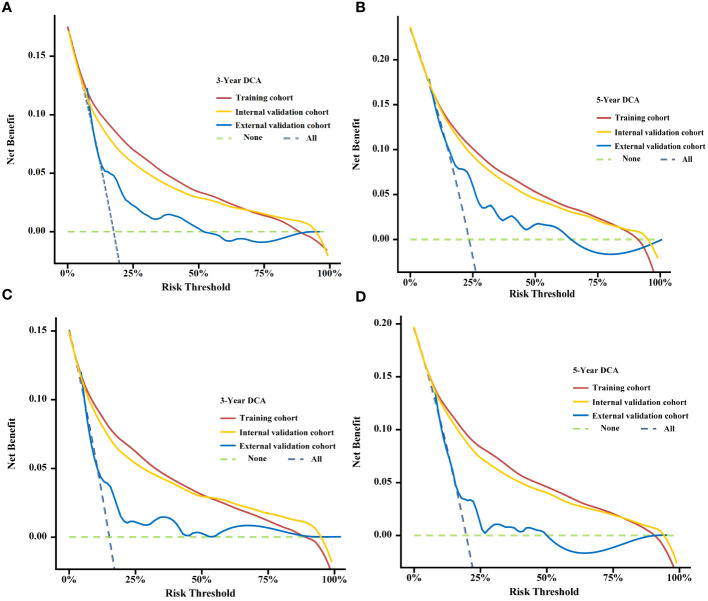
Decision curve analysis (DCA) curves for assessing clinical usefulness of the condition survival nomogram about 3- and 5-year OS **(A, B)**, 3- and 5-year BCSS **(C, D)** in training, Internal validation cohort, and External validation cohort.

By looking at the DCA curves, which demonstrated the net benefit of utilising the nomogram as a tool for triggering medical intervention vs. treating all or nothing, we may decide whether employing a model to help clinical decision-making will improve the outcomes of our patients. The results showed that the model’s net therapeutic benefit at 3 and 5 years was greater in the training and validation cohorts, falling within an appropriate threshold, suggesting that the nomogram has exceptional clinical efficacy (**Figure 7**).

Based on these findings, the nomogram technique established in this study is a good prognostic prediction tool for evaluating the probability of survival in patients with TNBC.

### 3.5 Kaplan–Meier survival analysis by nomogram risk category

The above investigations demonstrate the strong predictive power of the nomogram. The final stage involved assigning a risk score and developing a risk classification for each patient based on the nine characteristics of the nomogram.

**Figure 8:**
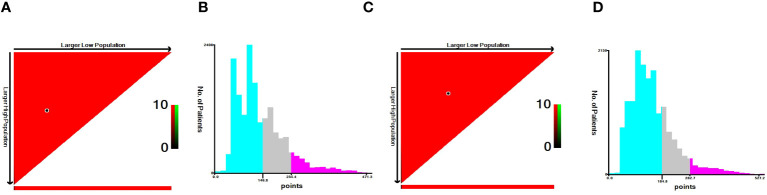
X-tile plots to identify the optimal risk score cutoff based on OS **(A, B)** and BCSS **(C, D)**.

**Figure 9:**
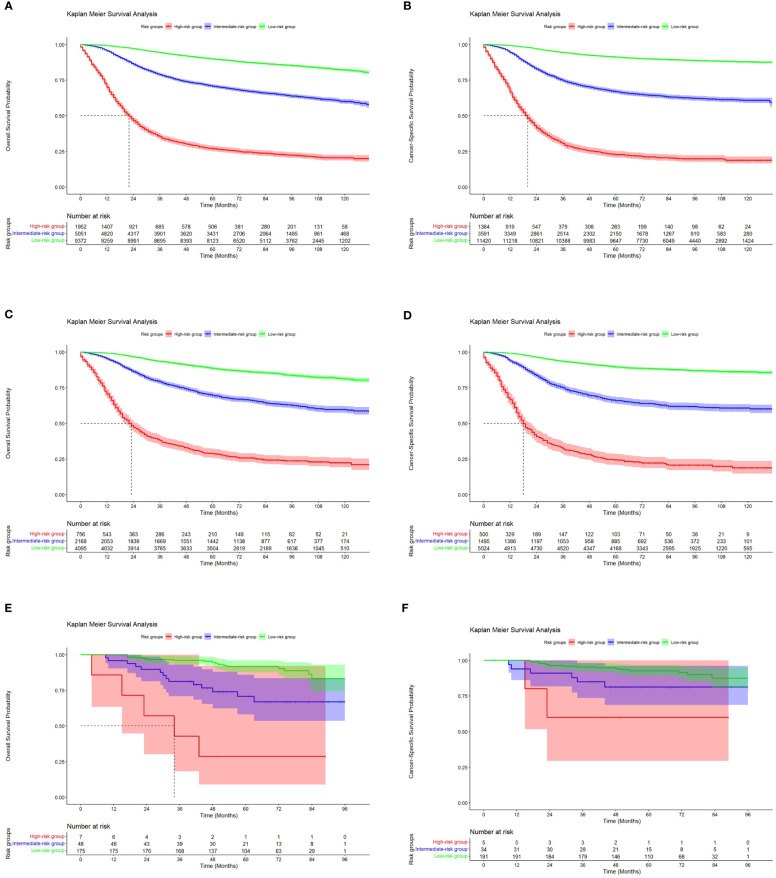
Kaplan-Meier curves of OS and BCSS for risk stratification in the training cohort **(A, B)**, the internal validation cohort **(C, D)** and the external validation cohort **(E, F)**.

Using X-tile software, we were able to calculate optimal cutoffs based on patient OS (**Figure 8**) and applied low risk (≤146 points), medium risk (>146 points and <235 points), and high risk (≥235 points) to all TNBC patients. Nomogram results from BCSS can also be used to classify patients into three categories: low risk (≤165 points), medium risk (>165 and <247 score), and high risk (≥247 points). According to Kaplan-Meier curve analysis, there were significant differences between high-risk and medium-risk patients in training (**Figures 9A, B**), internal validation ((**Figures 9C, D**), and external validation (**Figures 9E, F**) cohorts. The OS and BCSS of low-risk patients were significantly higher.

## 4 Discussion

Because TNBC is diverse and heterogeneous, the prognosis and course of treatment should be modified according to the patient’s physiological and clinical characteristics. This study aimed to develop a robust prognostic model with risk stratification that might predict survival for patients with TNBC and function as a roadmap for upcoming treatment interventions.

In the present study, 23,394 eligible individuals with TNBC were screened using the SEER database. To further screen and analyse the data, we used univariate, multivariate, and LASSO Cox regression analyses. This analysis revealed that independent prognostic factors, including age at diagnosis, marital status, grade, T stage, N stage, M stage, surgery, radiation, and chemotherapy, were significant predictors of OS and BCSS outcomes of patients with TNBC. These independent risk variables mostly agreed with clinical observations and were considered when creating the nomogram. The nomogram eliminated non-significant variables, such as race and histology, which helped doctors save time and effort by eliminating extraneous data collection.

However, between the training, internal validation, and external validation cohorts, the nomogram fared well in terms of accuracy and stability, and its performance for OS was higher than that of the TNM staging system’s C-index from the 7th edition of the AJCC (0.762 vs. 0.707, P < 0.01). A greater difference existed between the BCSS C-indices (0.793) and the median values reported in published prediction models (12). We used streamlined clinical data that were easily collected to create nomograms that are more useful and accurate in the real-world setting. The dynamic diagram was established to enhance the usability of the results.

Despite differences between The Affiliated Lihuili Hospital of Ningbo University’s external validation group and the SEER database’s training and validation groups, the hospital’s line diagram revealed satisfactory concordance in the external validation group. This chart aids medical professionals in identifying high-risk patients with poor survival rates, thereby improving clinical decision-making for the ongoing monitoring of TNBC patients.

Our nomogram demonstrated a strong ability to classify TNBC patients for risk, which can be applied to patient survival information as well as direct clinical decision-making and therapy allocation. We recommend that high-risk patients be classified as high-risk on the basis of a noma map and should be given intensive care and thorough follow-up because their prognosis is poor. When our hospital’s data was used to conduct the external validation of the BCSS, the median survival time of the patients could not be calculated. Indeed, the insufficient number of deaths in the single-centre sample, the standard treatment and active follow-up of patients with TNBC in our hospital, and the low Ki-67 index of the patients may be key contributing factors. In general, using this model, researchers can reduce patients’ psychological distress, improve treatment and follow-up adherence, and collect more valuable data for designing clinical trials.

Although similar work has been conducted by other investigators (13–15), this study has several notable strengths and innovations. Initially, we created a trustworthy nomogram to forecast BCSS and event-based OS in patients with TNBC and then included survival analysis and a clinical decision curve. Risk stratification can be used to a certain extent in the creation of individualised treatment regimens and prognostic assessments. Furthermore, by adding a Chinese cohort, we were able to increase the ethnic and geographic variety of the patients with TNBC in the study. The Chinese cohort also yielded satisfactory results when the model was externally validated. Finally, the developed prediction model can be used as a supplement or improvement to the TNM staging system, and we have created a web version for ease of use and calculation.

According to this study, patients with TNBC had an inverse relationship between the TNM stage and the histological grade. For clinical diagnosis and treatment of TNBC, determining the stage is essential. AJCC TNM staging is commonly utilised to determine prognoses in patients with breast cancer (8). According to the current study, patient prognosis worsened as the TNM stage increased, which is in line with other studies. However, the TNM staging approach has few variables, does not account for the patient’s particular state, pathological features, or prior treatment regimens, and cannot provide the patient with a personalised evaluation (16).

Additionally, our results imply that patients with TNBC older than 60 years have a very poor prognosis. Older patients with TNBC exhibit higher early mortality within the first 2 years after diagnosis compared with younger patients with TNBC (17, 18). However, in existing studies, non-breast cancer mortality was not excluded from the mortality figures, and some bias was present in judging the prognosis. Age had no discernible impact on BCSS in the current investigation.

However, the optimal surgical protocol for TNBC remains unclear. According to our study, patients who underwent surgery at the primary site experienced longer survival. Breast-conserving surgery (BCS) is the most used method to treat breast cancer. A growing trend toward minimally invasive and aesthetic treatments to achieve minimal trauma and optimal cosmetic results is occurring (19). Prior research has demonstrated that BCS is associated with a better prognosis than mastectomy for individuals with early-stage breast cancer (20, 21), and this is in line with the current study, which found that BCS was superior to mastectomy in terms of overall breast cancer survival and BCSS.

Right now, chemotherapy remains the mainstay of care for patients with TNBC (22, 23). Our investigation revealed significant differences in OS and BCSS between patients who received chemotherapy and those who did not. This suggests that active systemic chemotherapy should be administered in patients with TNBC. Currently, with the continuous development of multi-omics sequencing technology and an in-depth understanding of the biological behaviour of TNBC, new chemotherapeutic drugs (24, 25), antibody-conjugated drugs (26, 27), immunological checkpoint (PD-1 and PD-L1) inhibitors (28, 29), and poly ADP-ribosyl polymerase inhibitors (30) are gradually becoming more widely used in clinical practice, and the lack of chemotherapy protocols and medications no longer limits the treatment of TNBC. Furthermore, multigene assays, such as 70 genes (70-GS) (31) and 21 genes (21-RS) (32), are currently used in clinical practice to identify patients who are candidates for adjuvant chemotherapy. Nomograms paired with genome analysis may provide more relevant information for clinical decision-making.

In addition, our study showed that radiotherapy had less impact on OS and BCSS in patients with TNBC. Variations in the clinicopathological characteristics of individuals who received or did not receive radiation may be the cause of this observation. However, we did not take this prejudice into account in our comparisons. Therefore, more investigation is needed to ascertain how radiation affects the prognosis of individuals with TNBC.

Although our nomogram demonstrated excellent accuracy and clinical benefits through both internal and external validations, our study could be improved in several respects. First, although the SEER database contains data on chemotherapy and radiation therapy, including this information in the survival analysis was not recommended because of biases and missing factors related to treatment assignment in the SEER program. Second, no further research could be conducted because of insufficient data on the Ki-67 index, complications, body mass index, BRCA mutations, and family history. For instance, we were unable to create personalised estimates about the probability of recurrence because no information on local recurrence existed in the database. Furthermore, the constructed nomogram was only externally evaluated in one sample from China. Therefore, caution should be exercised when extrapolating the results to patients of different ethnic or geographic origins.

## 5 Conclusion

We developed and validated a dynamic prognostic nomogram for OS and BCSS in patients with TNBC based on nine independent prognostic indicators (age, marital status, grade, T stage, N stage, M stage, surgery, radiotherapy, and chemotherapy). Subsequently, we created a nomogram using various prognostic indicators to assess patient risk directly. The remarkable performance of our produced nomograms in the training and validation cohorts for predicting the 3- and 5-year OS and BCSS may help physicians in determining patient prognosis and creating individualised treatment plans.

## Data availability statement

The original contributions presented in the study are included in the article/**Supplementary Material**. Further inquiries can be directed to the corresponding author.

## Ethics statement

Ethical approval was not required for the study involving humans in accordance with the local legislation and institutional requirements. Written informed consent to participate in this study was not required from the participants or the participants’ legal guardians/next of kin in accordance with the national legislation and the institutional requirements.

## Author contributions

YQ: Writing – review & editing, Writing – original draft, Visualization, Validation, Supervision, Software, Resources, Project administration, Methodology, Investigation, Funding acquisition, Formal analysis, Data curation, Conceptualization. YC: Writing – original draft, Visualization, Supervision, Formal analysis, Data curation. HS: Writing – original draft, Supervision, Funding acquisition, Formal analysis, Data curation. SY: Writing – original draft, Supervision, Funding acquisition, Formal analysis, Data curation. JL: Writing – original draft, Formal analysis, Data curation. WW: Writing – review & editing, Writing – original draft, Visualization, Validation, Supervision, Software, Resources, Project administration, Methodology, Investigation, Funding acquisition, Formal analysis, Data curation, Conceptualization.
